# Circulating miRNA measurements are reflective of cholesterol-based changes in rainbow trout (*Oncorhynchus mykiss*)

**DOI:** 10.1371/journal.pone.0206727

**Published:** 2018-11-05

**Authors:** Tengfei Zhu, Geneviève Corraze, Elisabeth Plagnes-Juan, Sandrine Skiba-Cassy

**Affiliations:** INRA, Univ Pau & Pays Adour, E2S UPPA, UMR 1419, Nutrition Métabolisme Aquaculture, Saint Pée sur Nivelle, France; Universidade de Vigo, SPAIN

## Abstract

MicroRNAs (miRNAs) are a class of small non-coding RNAs which are known to posttranscriptionally regulate the expression of most genes in both animals and plants. Meanwhile, studies have shown that numbers of miRNAs are present in body fluids including the plasma. Despite the mode of action of these circulating miRNAs still remains unknown, they have been found to be promising biomarkers for disease diagnosis, prognosis and response to treatment. In order to evaluate the potential of miRNAs as non-invasive biomarkers in aquaculture, a time-course experiment was implemented to investigate the postprandial regulation of miRNAs levels in liver and plasma as well as the hepatic expression of genes involved in cholesterol metabolism. We showed that miR-1, miR-33a, miR-122, miR-128 and miR-223 were expressed in the liver of rainbow trout and present at detectable level in the plasma. We also demonstrated that hepatic expression of miR-1, miR-122 and miR-128 were regulated by feed intake and reached their highest levels 12 hours after the meal. Interestingly, we observed that circulating levels of miR-128 and miR-223 are subjected to postprandial regulations similar to that observed in their hepatic counterparts. Statistical correlations were observed between liver and plasma for miR-128 and miR-223 and between hepatic and circulating miR-122, miR-128 and miR-223 and expression of genes related to cholesterol synthesis and efflux or glucose phosphorylation. These results demonstrated that circulating miR-122, miR-128 and miR-223 are potential biomarkers of cholesterol metabolism in rainbow trout.

## Introduction

MicroRNAs (miRNAs) are a class of small non-coding RNAs of 19-24-mer nucleotides, mostly regulating gene expression at posttranscriptional level by mRNA decay or translational repression in animals and plants [1]. The high rate of miRNAs sequence conservation exhibited in many distantly related plant [2] and animal [3] species indicates their important functions in biological processes such as development, differentiation, proliferation, stress responses, apoptosis and oncogenesis [4–9].

Originating from independent transcription units, most miRNA genes are expressed under the control of their own promoters and regulatory sequences, nonetheless, others, located in gene introns are co-regulated with their host genes [4,5]. Initially transcribed by RNA polymerase II, a miRNA gene generates a long transcript called primary miRNA (pri-miRNA) in the nucleus which is then cleaved by a complex formed by RNase III enzyme Drosha and its binding partner DiGeorge syndrome critical region 8 (DGCR8) into a precursor miRNA (pre-miRNA). Following Exportin 5-dependent nuclear export, pre-miRNA is processed by RNase III enzyme Dicer and its interacting partner transactivation-responsive RNA-binding protein (TRBP) to generate a small double-stranded miRNA duplex which contains both the mature miRNA strand (guide strand) and its complementary strand (passenger strand). Finally, the miRNA duplex dissociates and the mature strand is carried by the Argonaute (AGO) protein forming the RNA-induced silencing complex (RISC), which decreases gene expression by mRNA decay or translational repression when perfect or imperfect complementarity occurs between miRNA and the targeted mRNA, respectively [10,11].

Pioneering works from Chim *et al*. found the presence of placental miRNAs in the maternal blood plasma [12] while, for the first time, Lawrie *et al*. suggested the potential utility of miRNAs in serum as non-invasive biomarkers for cancer diagnosis and prognosis [13]. Later, studies reported the presence of miRNAs in other body fluids, including plasma, tears, saliva, urine, breast milk, semen, amniotic fluid, colostrum, bronchial lavage, cerebrospinal fluid, peritoneal fluid, and pleural fluid [14–18], suggesting that circulating miRNAs may be involved in cell-to-cell communication [19]. It is also noteworthy that miRNAs are differently expressed in tissues as well as during developmental stages. For example, miR-122 is enriched in liver [20] whereas miR-133a and miR-133b are preferentially expressed in muscle [21]. miR-427 is reported to be transcribed only during blastula and gastrula stages of *Xenopus* development [22]. Since miRNAs were detected in various body fluids, it has been proposed to use circulating miRNAs as non-invasive biomarkers for disease diagnosis, prognosis and response to treatment [15,23]. Thus, the expression level of miR-141 in serum can thereby serve to distinguish prostate cancer patients from healthy controls [24] while it has also been shown that the expression level of miR-1 in serum is significantly higher in patients with acute myocardial infarction [25,26].

In contrast with mRNA, extracellular miRNAs are quite stable in nuclease- and protease-rich environment. Studies have shown that a majority of extracellular miRNAs can be protected from degradation by interacting with AGO proteins, which have a remarkable stability in extracellular environment [27,28]. Moreover, it has been demonstrated that miRNA stability is increased when miRNAs are engulfed in lipid vesicles, including apoptotic bodies, shedding vesicles, exosomes and high density lipoprotein (HDL) particles [29–33]. Although the selective release mechanism of circulating miRNAs as well as whether they could mediate cell-to-cell signaling remain unclear, they still appear as promising biomarkers thanks to their unique sequences, high stabilities in body fluids and tissue-specificities [34].

MiRNAs also play important roles in the development and physiological processes in fish [35–37]. First and most characterized in zebrafish [38][39][40][35], miRNAs are not much studied in fish compared to mammals. Beyond its importance in aquaculture, rainbow trout (*Oncorhynchus mykiss*) is also a model fish species worldwide studied for research in carcinogenesis, toxicology, comparative immunology, disease ecology, physiology and nutrition [41]. Therefore, studies of miRNAs in rainbow trout are of great interest for these scientific fields. In this context and during the last years, our laboratory and others initiated series of experiments in order to address the physiological importance of miRNAs in rainbow trout. In 2008, 14 miRNAs were identified and first reported in unfertilized eggs and early stage embryos of rainbow trout [42].Then, tissue specificities, targets predictions and genetic polymorphisms of miRNAs in rainbow trout were studied [43]. Subsequently, more miRNAs were identified in different rainbow trout tissues [44,45] and miRNAs involvement in development [46] and metabolic processes [47–49] were finally reported in rainbow trout.

Fishmeal and fish oil, the two traditional ingredients in fish feeds, are being replaced by plant ingredients in a much higher proportion to support the sustainable development of aquaculture [50]. Consequently, the nutrient composition of plant-based diet has greatly changed compared with diets mainly based on fishmeal and fish oil [51][52]. For example, cholesterol, which is normally mainly derived from animal ingredients, is almost absent in plant ingredients [53]. Previous studies on fish have shown that the expression of cholesterol related genes is affected when fishmeal and fish oil are replaced by plant ingredients [54–56]. Furthermore, when cholesterol is added to fish diet, plasma cholesterol level and the expression of genes related to cholesterol metabolism are also affected [57,58], indicating a significant effect of diet on cholesterol metabolism in fish.

In mammals, several miRNAs involved in the regulation of cholesterol or lipid metabolism have been identified, including miR-1, -33a, -122, -128 and -223. For instance, miR-1 is reported to repress *lxrα*, a master regulator of cholesterol homeostasis known to also target genes related to lipogenesis [59]. miR-33a is shown to act in concert with its host gene sterol regulatory element-binding protein (*srebp*) *-2* to target ATP-binding cassette transporter A1 (*abca1)* [60] and to regulate genes involved in cholesterol and lipid metabolism [61]. The inhibition of miR-122 in mice, which represents the most abundant miRNA in mammalian liver [20], revealed that it is a key regulator of cholesterol and fatty acid metabolism [62]. Accordingly with these results, we previously observed that miR-122 inhibition resulted in the decrease of cholesterol in plasma and the increase of gene expression involved in cholesterol degradation and excretion in rainbow trout [49]. Interestingly; miR-128 is shown to alter the expression of multiple genes involved in cellular cholesterol homeostasis through direct targeting of ATP-binding cassette transporters A1 and G1 mRNAs(*abca1* and *abcg1 respectively*) and retinoid X receptor (*rxrα*) [63]. Finally, the expression of miR-223 has been shown to inhibit cholesterol biosynthesis through the direct repression of sterol enzymes 3-hydroxy-3-methylglutaryl-CoA synthase (*hmgcs*) and methylsterol monooxygenase 1 (*sc4mol*) mRNAs and also to inhibit HDL-cholesterol uptake by scavenger receptor B1 (SR-B1) repression [64,65]. We also observed that miR-223 expression is suppressed, together with the expression of liver X receptor *α* (*lxrα*) in trout fed a plant-based diet while the expression of *srebp-2* is enhanced [56].

Therefore, we decided to evaluate if the abundance of circulating miRNAs could reflect the level of expression of these miRNAs in liver as well as those of related target genes. We undertook a parallel analysis of the hepatic expression and plasma abundance of miRNAs together with the hepatic expression of genes related to cholesterol metabolism in rainbow trout. The further determination of correlations between plasma and hepatic miRNAs as well as between circulating miRNAs and genes then helped us to evaluate the capacity of circulating miRNAs to be used as non-invasive biomarkers of tissue miRNA and gene expression.

## Materials and methods

### Ethics statement

Experiments were carried out in the INRA experimental facilities (UMR1419 Nutrition, Métabolisme, Aquaculture, Donzacq, France) authorized for animal experimentation by the French veterinary service which is the competent authority (A 64-495-1). Experiments were in strict accordance with EU legal frameworks related to the protection of animals used for scientific research (Directive 2010/63/EU) and according to the National Guidelines for Animal Care of the French Ministry of Research (decree n°2013–118, February 1st, 2013). Scientists in charge of the experimentation received a training and a personal authorization (N°B64 10 005). In agreement with the ethical committee “Comité d’Ethique Aquitaine Poissons Oiseaux” (C2EA-73), the present study does not need approval by a specific ethical committee since it implies only classical rearing practices with all diets formulated to cover the nutritional requirements of rainbow trout [33]. During this study, fish were daily monitored. If any clinical symptoms (i.e. morphological abnormality, restlessness or uncoordinated movements) were observed, fish were sedated by immersion in 10mg/L benzocaine solution and then euthanized by immersion in a 60mg/L benzocaine solution (anesthetic overdose) during 3 minutes.

### Sampling procedure

Rainbow trout (*Oncorhynchus mykiss*) (50g mean body weight) were reared in our experimental fish farm (INRA, Donzacq, France-permit n°A64-104-1) in open circuit tanks supplied with spring water at 17°C and under natural photoperiod. Fish were fed by hand twice a day with a commercial diet (Skretting, protein 42.5%, lipid 20.5%). At 3h, 8h, 12h, 16h and 24h after the last meal, six fish were anesthetized with benzocaine (30mg/L) and sacrificed by anesthetic overdose for sampling. Blood was removed from the caudal vein into syringes, rinsed with 10% EDTA and centrifuged (3000g, 5min). The recovered plasma was immediately frozen and kept at -80°C for miRNA expression and plasma metabolites analyses. Livers were dissected, weighted and 100 mg of sliced liver were sampled, put on absorbent paper to remove blood before being frozen in liquid nitrogen and kept at -80°C for hepatic gene and miRNA expression analyses.

### Plasma metabolites analysis

Plasma glucose, triglycerides and cholesterol concentrations were measured using commercial kits (BioMerieux, Marcy l’Etoile, France) adapted to microplate format, according to manufacturer’s recommendations.

### Hepatic gene expression analysis

For liver samples, total RNA was extracted as previously described [46] using Trizol reagent (Invitrogen, Carlsbad, CA, USA) according to manufacturer's instructions and were quantified by spectrophotometry (absorbance at λ = 260nm). The integrity of ARN samples was assessed by means of agarose gel electrophoresis. 1 μg of total RNA was used for cDNA synthesis. The SuperScript III RNaseH-reverse transcriptase kit (Invitrogen) with oligo dT random primers (Promega, Charbonniéres, France) was used to synthesize cDNA (n = 6). The primer sequences used for Reverse Transcription-quantitative Polymerase Chain Reaction RT-qPCR analyses are listed in the Table 1. RT-qPCR gene expression analyses were focused on genes involved in cholesterol metabolism, including ATP-binding cassette transporter A1 (*abca1*), ATP-binding cassette transporter G5 (*abcg5*), ATP-binding cassette transporter G8 (*abcg8*), Acyl-CoA cholesterol acyltransferase 1 (*acat1*), Acyl-CoA cholesterol acyltransferase 2 (*acat2*), Lanosterol 14α-demethylase (*cyp51*), cholesterol 7α-hydroxylase (*cyp7a1*), 7-dehydrocholesterol reductase (*dhcr7*), HMG-CoA reductase (*hmgcr*), HMG-CoA synthase (*hmgcs*), liver X receptor *α* (*lxrα*), sterol regulatory element-binding protein 2 (*srebp-2*) and UDP glycuronosyltransferase 1 A3 (*ugt1a3*); lipogenesis, including fatty acid synthase (*fas*) and sterol regulatory element-binding protein 1c (*srebp-1c*) and glycolysis, glucokinase (*gck*).

**Table 1:** Sequence of the primers in qRT-PCR for gene expression analysis.

	Forward primer	Reverse primer	Genoscope^1^ or Genbank accession number	
			Paralogue 1	Paralogue 2
*abca1*	CAGGAAAGACGAGCACCTT	TCTGCCACCTCACACACTTC	GSONMG00078741001	GSONMG00074045001
*abcg5*	CACCGACATGGAGACAGAAA	GACAGATGGAAGGGGATGAA	GSONMG00075025001	/
*abcg8*	GATACCAGGGTTCCAGAGCA	CCAGAAACAGAGGGACCAGA	GSONMG00075024001	/
*acat1*	GGCAAGCCTGATGTGGTAGT	ACCGTGCCATTCTCCTTCTG	GSONMG00033938001 D2OM_LOC100702912.1.1	/
*acat2*	TGCTTGTTGTCCCTGGGTTT	GTGTGGCTGTGACGTGTTTC	GSONMG00054446001 D2OM_THIC.1.1	/
*cyp51*	CCCGTTGTCAGCTTTACCA	GCATTGAGATCTTCGTTCTTGC	GSONMG00031182001	GSONMG00044416001
*cyp7a1*	ACGTCCGAGTGGCTAAAGAG	GGTCAAAGTGGAGCATCTGG	GSONMG00066448001 AB675933.1	GSONMG00037174001 AB675934.1
*dhcr7*	GTAACCCACCAGACCCAAGA	CCTCTCCTATGCAGCCAAC	GSONMG00025402001	GSONMG00039624001
*fas*	TGATCTGAAGGCCCGTGTCA	GGGTGACGTTGCCGTGGTAT	GSONMG00062364001	
*gck*	GCACGGCTGAGATGCTCTTTG	GCCTTGAACCCTTTGGTCCAG	GSONMG00033781001	GSONMG00012878001
*hmgcr*	GAACGGTGAATGTGCTGTGT	GACCATTTGGGAGCTTGTGT	GSONMG00016350001	/
*hmgcs*	AGTGGCAAAGAGAGGGTGTG	TTCTGGTTGGAGACGAGGAG	GSONMG00010243001 D2OM_LOC100712075.1.1	/
*lxrα*	TGCAGCAGCCGTATGTGGA	GCGGCGGGAGCTTCTTGTC	GSONMG00014026001	GSONMG00064070001
*srebp-1c*	CATGCGCAGGTTGTTTCTT	GATGTGTTCGTGTGGGACTG	XM_021624594.1	
*srebp-2*	TAGGCCCCAAAGGGATAAAG	TCAGACACGACGAGCACAA	GSONMG00039651001	GSONMG00061885001
*ugt1a3*	CCACCAGCAAGACAGTCTCA	CAACAGCACAGTGGCTGACT	GSONMG00035844001	/

^1^ https://www.genoscope.cns.fr/trout

Primers were designed to coamplify different paralogs of the same gene.

RT-qPCR assays were performed on the Roche LightCycler 480 II system (Roche Diagnostics, Neuilly sur Seine, France). The assays were carried out using a reaction mix of 6 μL per sample containing 2 μL of 76 times diluted cDNA, 0.24 μL of each primer (10 μM), 3 μL of LightCycler 480 SYBR Green I Master mix and 0.52 μL DNAse/RNAse free water (5 Prime GmbH, Hamburg, Germany). The PCR protocol was initiated at 95°C for 10 min for initial denaturation of the cDNA and hot-start Taq-polymerase activation, followed by 45 cycles of a three-step amplification program (15s at 95°C, 10s at melting temperature Tm (60–65°C), 15s at 72°C), according to the primer set used. Melting curves were systematically monitored (5 s at 95°C, 1 min at 65°C, temperature gradient at 0.11°C/s from 65 to 97°C) at the end of the last amplification cycle to confirm the specificity of the amplification reaction. Each PCR assay included replicate samples (duplicate of reverse transcription and PCR amplification) and negative controls (RT- and cDNA-free samples, respectively). Relative quantification of target gene expression was determined using the E-Method from the LightCycler 480 software (version SW 1.5; Roche Diagnostics). The NORMA-Gene algorithm not using housekeeping genes was used for the normalization by calculating the average deviation from a sample to the mean of specific genes [66] as the expression of the 3 reference genes tested (β-actin, EF1, 18 S) was not consistent between different sampling time points. In all cases, PCR efficiency (E) measured by the slope of a standard curve with serial dilutions of cDNA ranged between 1.8 and 2.0.

### Hepatic miRNA expression analysis

Expression of hepatic microRNAs (miR-1-3p, miR-33a-5p, miR-122-5p, miR-128-3p and miR-223-3p) was assessed by means of the TaqMan Advanced miRNA Assays (A25576, Applied Biosystems, ThermoFisher) according to manufacturer’s instructions. Synthetic cel-miR-39 mimic (miScript miRNA Mimics, QIAGEN) was added to each RNA sample and used as an exogenous control for normalization. It exhibited a constant level in liver samples at the different sampling time points. Briefly, 80 ng of total RNA were used for the poly(A) tailing, ligation and reverse transcription reactions to synthesize the cDNA of all miRNAs followed by a pre-amplification step. qPCR was then performed in a reaction mix of 6 μL containing 2 μL cDNA (200 times diluted), 2.67 μL 2X Fast Advanced Master mix (Applied Biosystems, USA), 0.27 μL TaqMan Advanced miRNA Assay (20X) (Applied Biosystems, USA) and 1.06 μL DNAse/RNAse free water (5 Prime GmbH, Hamburg, Germany). Sequences of the miRNA probes were listed in Table 2.

**Table 2:** Sequences of probes used for TaqMan advanced miRNA assays.

Assay	Assay ID	Probe sequence
miR-1-3p	477820_mir	TGGAATGTAAAGAAGTATGTAT
miR-33a-5p	478347_mir	GTGCATTGTAGTTGCATTGCA
miR-39-3p	478293_mir	UCACCGGGUGUAAAUCAGCUUG
miR-122-5p	477855_mir	TGGAGTGTGACAATGGTGTTTG
miR-128-3p	477892_mir	TCACAGTGAACCGGTCTCTTT
miR-223-3p	477983_mir	TGTCAGTTTGTCAAATACCCCA

The PCR protocol was initiated at 95°C for 20s for initial denaturation of the cDNA and the enzyme activation, followed by 50 cycles of a 2 steps amplification program (3s at 95°C for denaturation, 30s at 60°C for annealing). Each PCR assay included replicates for each sample (duplicates of reverse transcription and PCR amplification) and also negative controls (reverse transcriptase free and RNA free samples). Relative quantification of the target miRNAs was determined using the E-Method from the LightCycler 480 software (version SW 1.5; Roche Diagnostics). PCR efficiency measured by the slope of a standard curve with serial dilutions of miRNA cDNA ranged between 1.9 and 2.1.

### Plasmatic miRNA expression analysis

miR-1-3p, miR-33a-5p, miR-122-5p, miR-128-3p and miR-223-3p were detected in plasma samples by means of the TaqMan Advanced miRNA Assays (A25576, Applied Biosystems, ThermoFisher). Synthetic cel-miR-39 mimic (miScript miRNA Mimics, QIAGEN) was added and used as an exogenous control for normalization and showed constant levels in plasma samples.

Total RNA was extracted from plasma samples with the TRIzol LS reagent (Life Technologies, Carlsbad, CA, USA) according to manufacturer's instructions and was quantified by spectrophotometry (absorbance at λ = 260nm). The cDNA synthesis of all miRNAs and the following qPCR steps were performed in the same way as those previously described for hepatic miRNA expression analysis. PCR efficiency measured by the slope of a standard curve with serial dilutions of miRNA cDNA was nearly 2.0.

### Statistical analysis

Results are expressed as means ± SD. Statistical analyses were carried out using one-way ANOVA to detect significant differences, followed by a Turkey test for post hoc analysis. Normality was assessed using the Shapiro-Wilk test, while homogeneity of variance was determined using Levene’s test. In cases where data were nonparametric or not homoscedastic, data transformations, such as logarithms, were used to meet ANOVA criteria. For all statistical analysis, the level of significance was set at *P*<0.05. Pearson correlation coefficients were calculated based on data of normalized genes or miRNA expression calculated by the E-Method from the LightCycler 480 software (version SW 1.5; Roche Diagnostics). All statistical analyses were performed using R software [67].

## Results

The complete dataset of the study is presented within the manuscript and in its Supporting Information files (S1 File).

### Postprandial cholesterol, glucose and triglyceride levels in the plasma of rainbow trout

**Fig 1:**
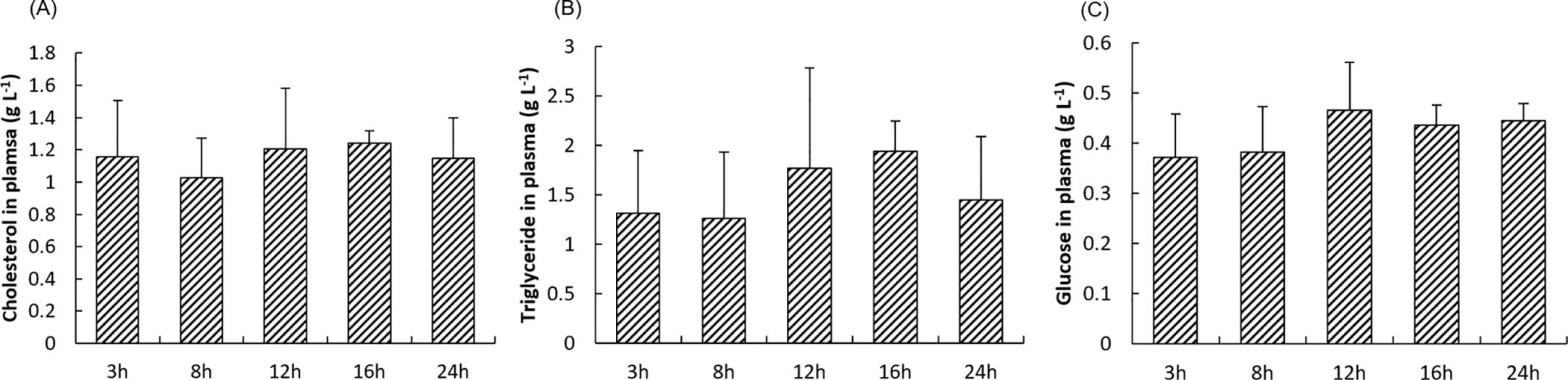
**Postprandial levels of cholesterol (A), triglycerides (B) and glucose (C) in plasma of rainbow trout at 3h, 8h, 12h, 16h and 24h after the meal.** Values are means (n = 9), with their standard deviations represented by vertical bars.

No significant changes were found for cholesterol, triglycerides and glucose levels between plasma samples collected at different time points after the meal (Fig 1).

### Postprandial hepatic gene expression

**Fig 2:**
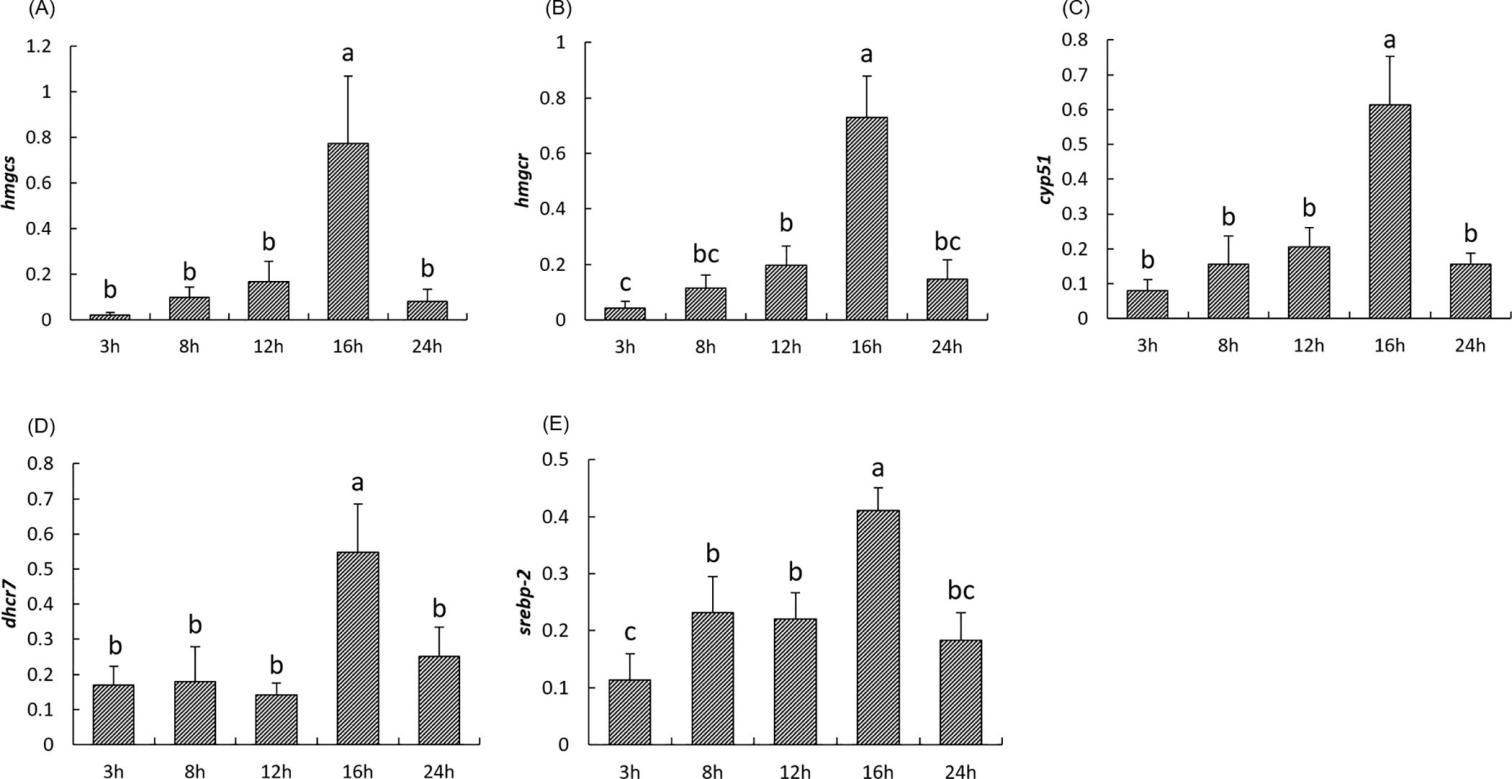
**Postprandial expression of genes involved in cholesterol synthesis including *hmgcs (A)*, *hmgcr (B)*, *cyp51 (C)*, *dhcr7 (D)*, *srebp-2 (E)* in the liver of rainbow trout at 3h, 8h, 12h, 16h and 24h after the meal.** The NORMA-Gene method was used for the normalization. Values are means (n = 6), with their standard deviations represented by vertical bars. ^a, b, c^ Mean values with unlike letters were significantly different (*P*<0.05; One-way analysis of variance, Tukey’s test).

The expression of the four main genes involved in cholesterol synthesis (*hmgcr*, *hmgcs*, *cyp51* and *dhcr7*) and the transcription factor *srebp-2* significantly increased reaching their highest level of expression 16h after the meal (*P<0*.*05*). The expression of these genes returned to basal level 24h after the meal with similar expressions compared with samples collected 3h after the meal (Fig 2). Pearson correlation analysis revealed that the expression of *srebp-2* and its four target genes (*hmgcr*, *hmgcs*, *cyp51*, *dhcr7*) were all positively correlated (Table 3).

**Table 3:** Correlations between gene expressions involved in cholesterol metabolism of rainbow trout.

Pearson value		Cholesterol synthesis					Bile acid synthesis		Cholesterol efflux				Esterification		Lipogenesis		
n = 29		*hmgcs*	*hmgcr*	*cyp51*	*dhcr7*	*srebp-2*	*cyp7a1*	*ugt1a3*	*abcg5*	*abcg8*	*abca1*	*lxr*	*acat1*	*acat2*	*fas*	*srebp-1c*	*gck*
Cholesterol synthesis	*hmgcs*		0.942**	0.957**	0.870**	0.830**	-0.1573	0.1301	0.1857	0.565**	-0.1408	-0.3116	0.433*	0.806**	0.848**	0.793**	0.432*
	*hmgcr*			0.979**	0.875**	0.863**	-0.1811	0.361*	0.2173	0.667**	-0.1816	-0.3186	0.528**	0.776**	0.870**	0.710**	0.517**
	*cyp51*				0.898**	0.895**	-0.1841	0.2825	0.1692	0.615**	-0.1133	-0.3047	0.487**	0.775**	0.841**	0.729**	0.471**
	*dhcr7*					0.742**	-0.0174	0.3039	0.3497	0.696**	-0.2184	-0.1253	0.501**	0.566**	0.840**	0.641**	0.518**
	*srebp-2*						-0.3235	0.2252	-0.0283	0.533**	0.0893	-0.2301	0.569**	0.711**	0.701**	0.762**	0.411*
Bile acid synthesis	*cyp7a1*							0.2908	0.549**	0.247	-0.05	0.409*	0.0123	-0.2376	-0.1453	-0.2186	0.2128
	*ugt1a3*								0.493**	0.583**	-0.2284	0.061	0.491**	0.0115	0.377*	0.0189	0.630**
Cholesterol efflux	*abcg5*									0.473**	-0.373*	0.431*	0.449*	0.0093	0.3446	0.1457	0.532**
	*abcg8*										-0.2559	-0.1003	0.450*	0.289	0.620**	0.2953	0.619**
	*abca1*											0.0935	-0.0589	-0.1452	-0.373*	-0.1535	-0.3369
	*lxr*												0.2506	-0.3376	-0.3017	-0.1498	-0.0056
Esterification	*acat1*													0.393*	0.508**	0.585**	0.470**
	*acat2*														0.622**	0.760**	0.2371
Lipogenesis	*fas*															0.757**	0.603**
	*srebp-1c*																0.3612
	*gck*																

P-values

* P <0.05

** P <0.01

Pearson correlation coefficient larger than zero represents a positive correlation, while Pearson correlation coefficient less than zero represents a negative correlation.

**Fig 3:**
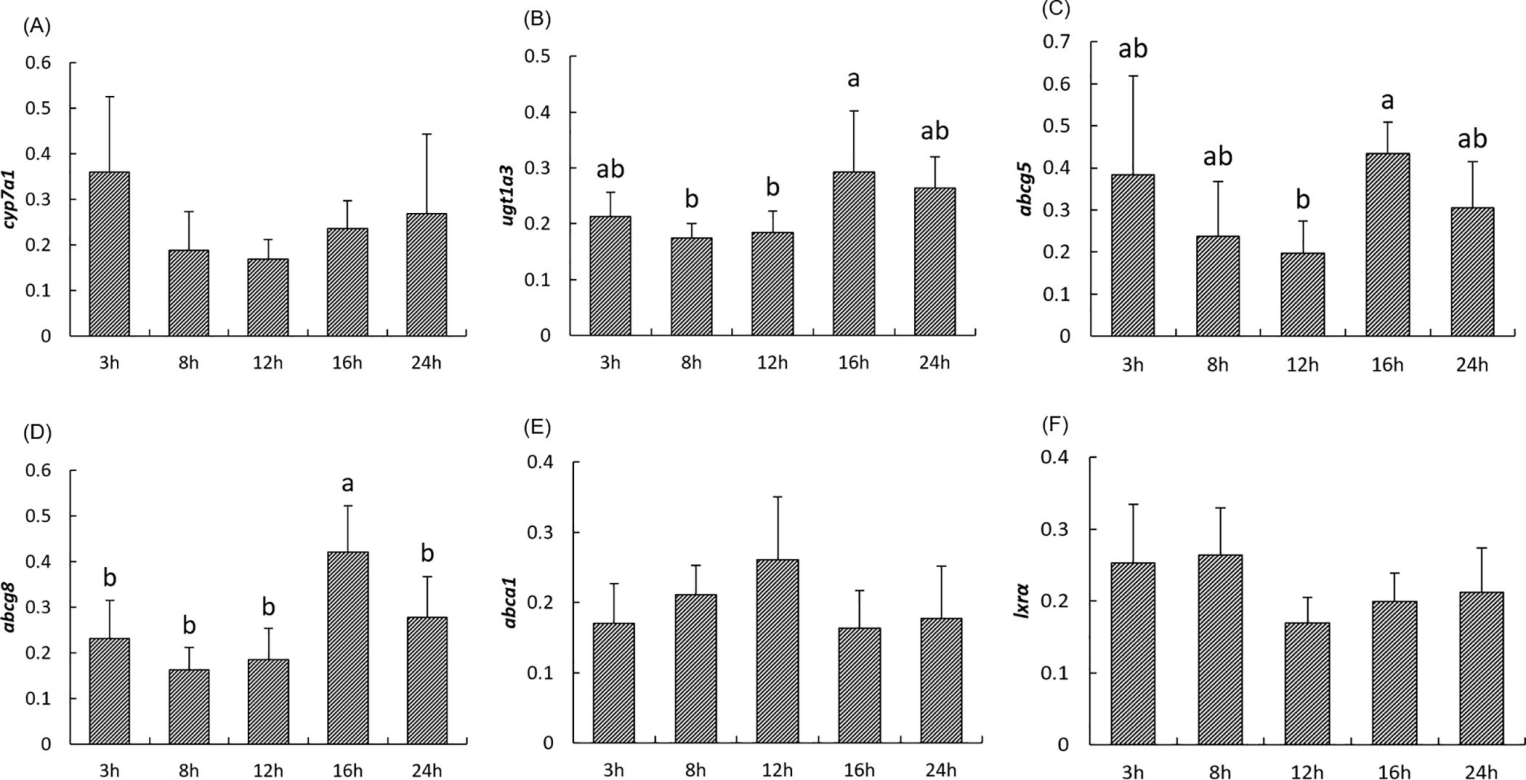
**Postprandial expression of genes involved in cholesterol efflux including *cyp7a1 (A)*, *ugt1a3 (B)*, *abcg5 (C)*, *abcg8 (D)*, *abca1(E) lxrα (F)* in the liver of rainbow trout at 3h, 8h, 12h, 16h and 24h after the meal.** The NORMA-Gene method was used for the normalization. Values are means (n = 6), with their standard deviations represented by vertical bars. ^a, b^ Mean values with unlike letters were significantly different (*P*<0.05; One-way analysis of variance, Tukey’s test).

As observed for genes involved in cholesterol synthesis, *ugt1a3* responsible for the glucuronidation of bile acid, *abcg5* and *abcg8*, implicated in biliary excretion of cholesterol, exhibited highest level of expression 16h after the meal (*P<0*.*05*) (Fig 3). However, no significant postprandial changes were found for the expression of *cyp7a1*which is the key enzyme of bile acid synthesis, *abca1* which mediates the efflux of cholesterol and phospholipids to lipid-poor apolipoproteins to form nascent high-density lipoproteins and the transcription factor *lxrα* (*P>0*.*05*) (Fig 3). Meanwhile, the postprandial expression of *ugt1a3*, *abcg5*, and *abcg8* were positively correlated with each other and the expression of *lxrα* was found to be positively correlated with *cyp7a1* and *abcg5* as well (Table 3). The expression of *abcg8* positively correlated with all genes involved in cholesterol synthesis, while *abcg5* correlated with all genes involved in cholesterol efflux in a positive way except for *abca1* in a negative way (Table 3). The expression of *abcg5* was positively correlated with plasma cholesterol and triglycerides whereas an opposite correlation was found between plasma cholesterol and triglycerides and *abca1* (Table 4).

**Table 4:** Correlations between hepatic gene expressions involved in cholesterol metabolism and plasma parameters of rainbow trout.

Pearson value	Cholesterol synthesis					Bile acid synthesis		Cholesterol efflux				Esterification		Lipogenesis		Glycogenesis
n = 30	*hmgcs*	*hmgcr*	*cyp51*	*dhcr7*	*srebp-2*	*cyp7a1*	*ugt1a3*	*abcg5*	*abcg8*	*abca1*	*lxr*	*acat1*	*acat2*	*Fas*	*srebp-1c*	*gck*
CHOL	0.160	0.140	0.062	0.141	-0.182	0.167	0.127	0.571**	0.183	-0.477**	-0.050	0.076	0.087	0.374*	0.151	0.051
TG	0.249	0.254	0.173	0.108	-0.003	-0.067	0.134	0.401*	0.072	-0.517**	-0.136	0.163	0.285	0.480**	0.337	0.188
GLU	0.157	0.182	0.178	0.134	0.088	0.176	0.153	0.315	0.197	0.219	-0.032	-0.026	-0.144	0.166	0.023	0.180

P-values

* *P*<0.05

** P<0.01.

Pearson correlation coefficient larger than zero represents a positive correlation, while Pearson correlation coefficient less than zero represents a negative correlation.

**Fig 4:**
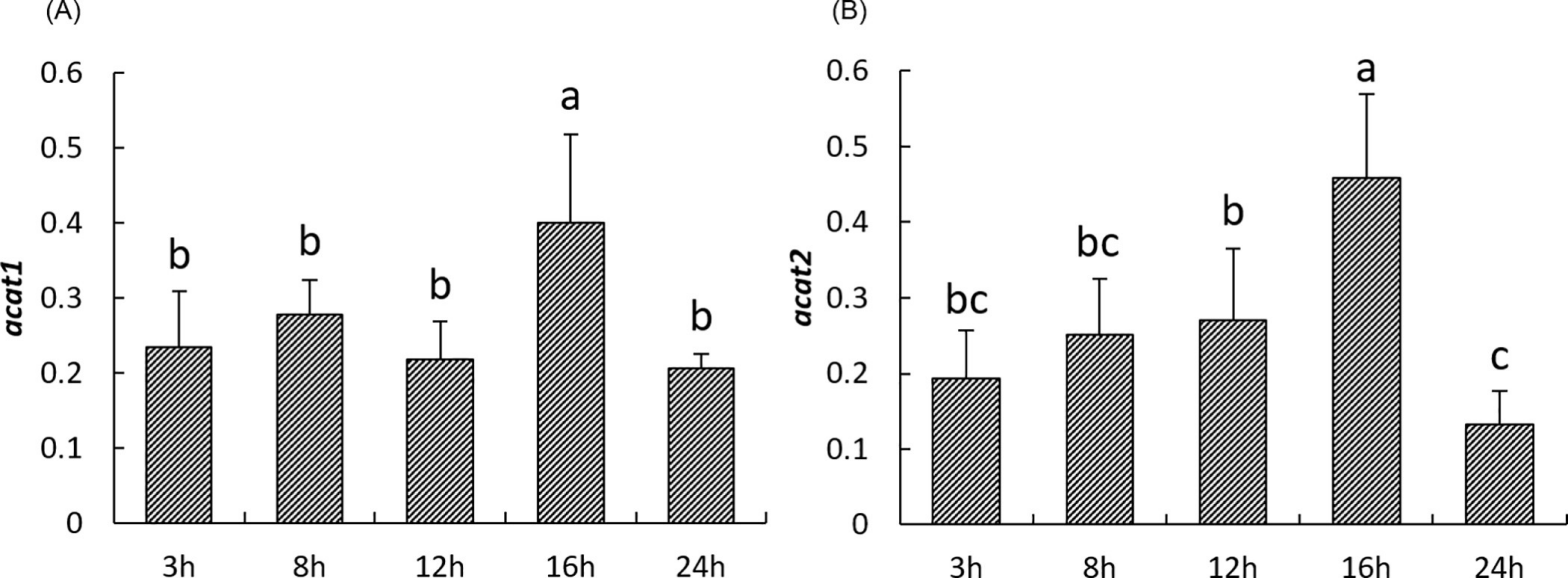
**Postprandial expression of genes involved in cholesterol esterification including *acat1(A)*, *acat2 (B)* in the liver of rainbow trout at 3h, 8h, 12h, 16h and 24h after the meal.** The NORMA-Gene method was used for the normalization. Values are means (n = 6), with their standard deviations represented by vertical bars. ^a, b, c^ Mean values with unlike letters were significantly different (*P*<0.05; One-way analysis of variance, Tukey’s test).

Regarding genes involved in cholesterol esterification, the highest expression of *acat1* and *acat2* was observed 16h after the meal (*P<0*.*05*) and then decreased to their basal level 24h after feeding (Fig 4). Both *acat1* and *acat2* were positively correlated with each other but also with genes involved in cholesterol synthesis. In addition, *acat1* was found to positively correlate with *ugt1a3*, *abcg5*, and *abcg8*, genes involved in cholesterol efflux (Table 3).

**Fig 5:**
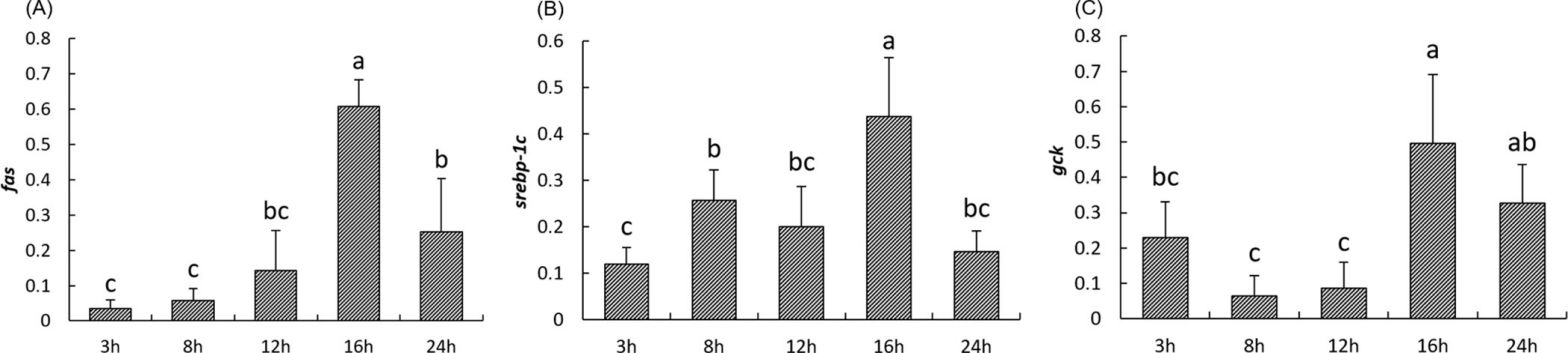
**Postprandial expression of genes involved in lipogenesis including *fas (A)*, *srebp-1c (B)* and *gck* (C) in the liver of rainbow trout at 3h, 8h, 12h, 16h and 24h after the meal.** The NORMA-Gene method was used for the normalization. Values are means (n = 6), with their standard deviations represented by vertical bars. ^a, b, c^ Mean values with unlike letters were significantly different (*P*<0.05; One-way analysis of variance, Tukey’s test).

Expression of the lipogenic gene *fas* and its transcription factor *srebp-1c* significantly increased 16h after the meal (*P<0*.*05*) and then decreased between 16h and 24h. *gck* which is considered as the first enzyme of lipogenesis that induces glucose phosphorylation showed a similar pattern (Fig 5). *fas* was positively correlated with *srebp-1c* and genes involved in cholesterol synthesis, efflux and esterification (Table 3) as well as with the level of cholesterol and triglycerides in the plasma (Table 4).

### Postprandial miRNAs expression

**Fig 6:**
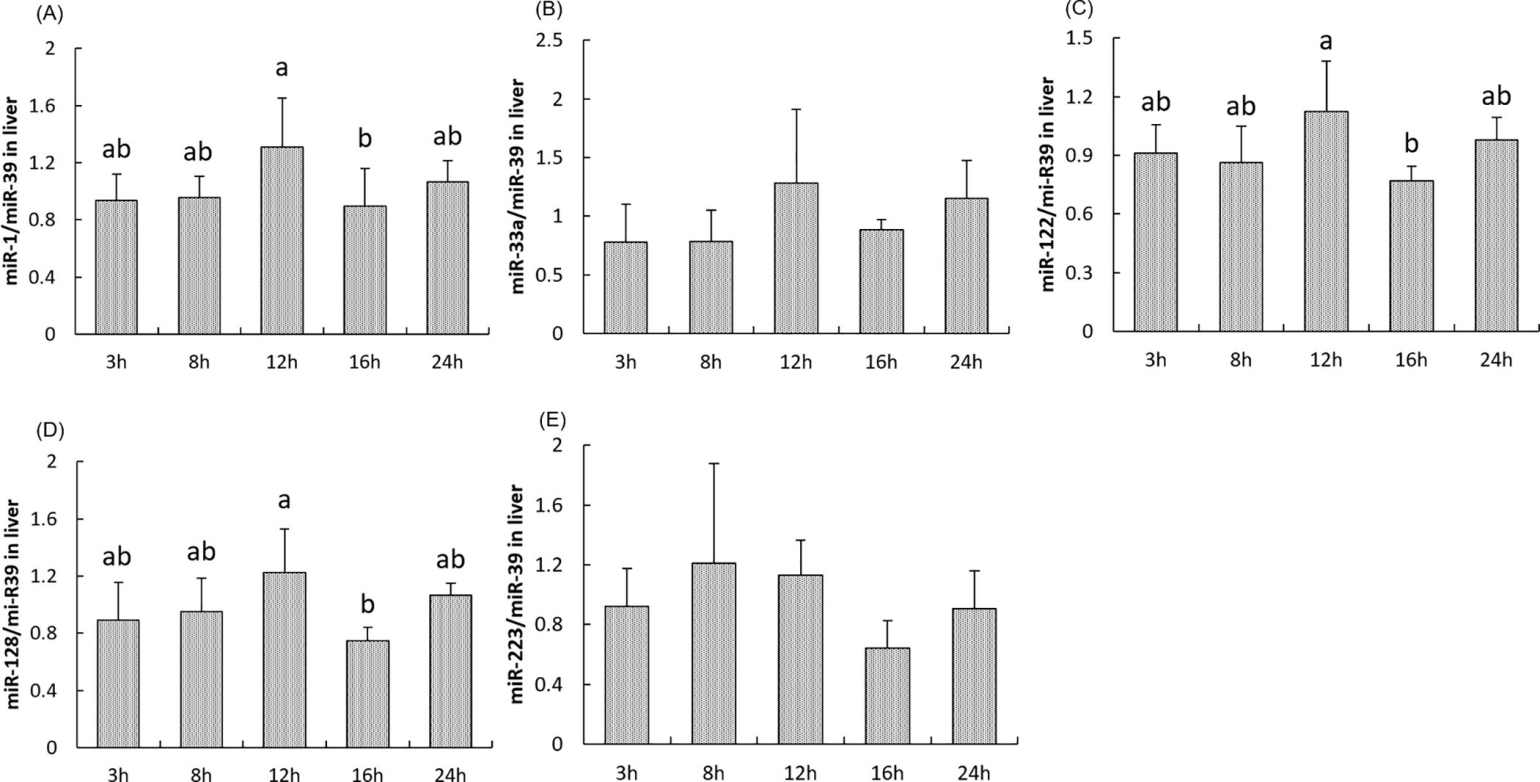
**Postprandial expression of miRNAs involved in cholesterol metabolism miR-1(A), miR-33a (B), miR-122(C), miR-128(D), and miR-223(E) in the liver of rainbow trout at 3h, 8h, 12h, 16h and 24h after the meal.** Indicated miR expression values were normalized to miR-39 levels. Values are means (n = 6), with their standard deviations represented by vertical bars. ^a, b^ Mean values with unlike letters were significantly different (*P*<0.05; One-way analysis of variance, Tukey’s test).

The highest and lowest levels of hepatic miR-1, miR-122 and miR-128 were observed 12h and 16h after the meal, respectively (*P<0*.*05*). No significant changes were found for miR-33a and miR-223 during the postprandial period (Fig 6).

**Fig 7:**
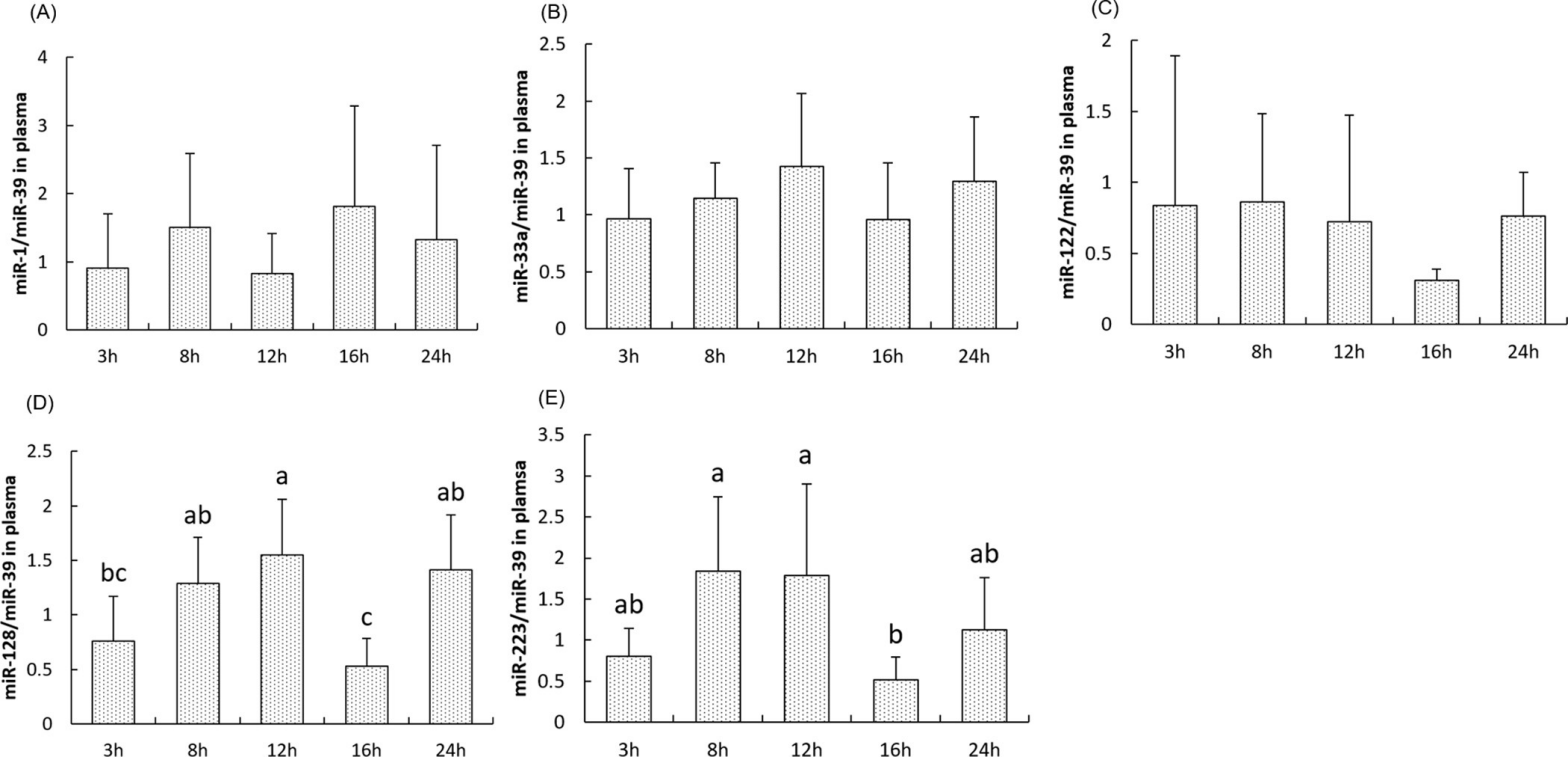
**Postprandial expression of miRNAs involved in cholesterol metabolism miR-1(A), miR-33a (B), miR-122(C), miR-128(D), and miR-223(E) in the plasma of rainbow trout at 3h, 8h, 12h, 16h and 24h after the meal.** Expression values were normalized to miR-39 levels. Values are means (n = 6), with their standard deviations represented by vertical bars. ^a, b, c^ Mean values with unlike letters were significantly different (*P*<0.05; One-way analysis of variance, Tukey’s test).

In the meantime, we analyzed the miRNA abundance in plasma of the same fish. Whereas no postprandial variation was observed for miR-1, miR-33 and miR-122 in plasma samples, our results indicated a significant rise of miR-128 abundance in the plasma at 12h of the postprandial time course before to significantly decrease to its lowest abundance 4h after. Interestingly and despite the absence of difference of miR-233 in postprandial livers, circulating miR-223 peaked 8h after the meal and remained stable for 4 more hours before to decrease to its lowest level 16h after the meal (*P<0*.*05*), (Fig 7).

### Correlations between plasmatic parameters and miRNAs in liver and plasma

The expression of miR-33a in liver was positively correlated with the level of cholesterol and triglycerides in plasma (*P<0*.*01*). On the other hand, circulating miR-223 negatively correlated with the level of cholesterol and triglycerides (*P<0*.*05*). Meanwhile, the plasma miR-122 and glucose levels showed positive correlation between each other (*P<0*.*05*) (Table 5).

**Table 5:** Correlations of plasmatic parameters, miRNAs expression in the liver, and miRNAs level in the plasma of rainbow trout.

Pearson value	n = 30	n = 30	n = 30	n = 30	n = 30	n = 30	n = 30	n = 29	n = 30	n = 30
	L-miR-1	L-miR-33a	L-miR-122	L-miR128	L-miR-223	P-miR-1	P-miR-33a	P-miR-122	P-miR-128	P-miR-223
CHOL	0.229	0.618**	-0.045	0.045	-0.189	0.026	0.006	0.255	-0.119	-0.388*
GLU	0.285	0.199	0.157	0.126	-0.256	-0.056	0.357	0.369*	0.321	0.239
TG	0.213	0.671**	-0.007	0.084	-0.163	0.126	0.091	0.127	-0.093	-0.401*

n = 30 for miRNAs in plasma except for miR-122 (n = 29).

P-values

* *P*<0.05

** *P*<0.01.

Pearson correlation coefficient larger than zero represents a positive correlation, while Pearson correlation coefficient less than zero represents a negative correlation.

### Correlations between hepatic genes and miRNAs in liver and plasma

Regarding the expression of miRNAs in the liver, miR-122, miR-128 and miR-223 presented negative correlations with genes involved in cholesterol synthesis (*P<0*.*05*), especially the miR-128 whose expression was correlated with four cholesterol synthetic genes, namely *hmgcs*, *hmgcr*, *cyp51 and dhcr7*. Additionally, miR-128 expression was also negatively correlated with *acat2*, which is involved in cholesterol esterification (*P<0*.*05*). The expression of miR-223 was negatively correlated with *abcg8* involved in cholesterol efflux (*P<0*.*05*) as well as *gck* involved in glycolysis (*P<0*.*01*) (Table 6).

**Table 6:** Correlations of hepatic gene expression involved in cholesterol metabolism, miRNAs expression in the liver, and miRNAs level in the plasma of rainbow trout.

Pearson Value		Cholesterol synthesis					Bile acid synthesis		Cholesterol efflux				Esterification		Lipogenesis		Glycogenesis
		*hmgcs*	*hmgcr*	*cyp51*	*dhcr7*	*srebp-2*	*cyp7a1*	*ugt1a3*	*abcg5*	*abcg8*	*abca1*	*lxr*	*acat1*	*acat2*	*fas*	*srebp-1c*	*gck*
n = 30	L-miR-1	-0.134	-0.191	-0.196	-0.249	-0.180	0.046	-0.261	-0.179	-0.197	0.223	-0.128	-0.191	-0.268	-0.084	-0.018	-0.331
n = 30	L-miR-33a	-0.094	-0.075	-0.123	-0.146	-0.270	-0.112	-0.050	0.126	-0.175	-0.248	-0.178	-0.120	-0.130	0.222	0.051	-0.061
n = 30	L-miR-122	-0.378*	-0.345	-0.342	-0.408*	-0.301	-0.086	-0.192	-0.202	-0.265	0.083	0.050	-0.257	-0.308	-0.275	-0.298	-0.240
n = 30	L-miR-128	-0.439*	-0.385*	-0.376*	-0.439*	-0.306	-0.157	-0.201	-0.215	-0.339	0.080	-0.009	-0.218	-0.364*	-0.250	-0.249	-0.322
n = 30	L-miR-223	-0.415*	-0.336	-0.292	-0.303	-0.16	-0.249	-0.283	-0.348	-0.377*	0.135	0.162	-0.035	-0.268	-0.351	-0.204	-0.501**
n = 29	P-miR-1	0.264	0.331	0.287	0.308	0.247	-0.188	0.033	-0.113	0.210	-0.273	-0.052	0.099	0.173	0.232	0.147	0.321
n = 29	P-miR-33a	-0.174	-0.132	-0.166	-0.160	-0.138	-0.050	-0.232	-0.071	-0.149	0.043	0.208	-0.117	-0.212	-0.071	-0.070	0.016
n = 29	P-miR-122	-0.328	-0.372*	-0.424*	-0.329	-0.431*	0.361	-0.169	0.378*	-0.059	0.054	0.284	-0.076	-0.376*	-0.261	-0.190	-0.100
n = 29	P-miR-128	-0.438*	-0.421*	-0.408*	-0.335	-0.310	-0.136	-0.331	-0.239	-0.355	0.310	0.188	-0.258	-0.459*	-0.326	-0.289	-0.327
n = 29	P-miR-223	-0.334	-0.305	-0.252	-0.266	-0.109	-0.292	-0.465*	-0.408*	-0.414*	0.456*	0.120	-0.173	-0.287	-0.383*	-0.146	-0.452*

n = 30 for miRNAs in liver, n = 29 for miRNAs in plasma.

p-values

* *P*<0.05

** *P*<0.01.

Pearson correlation coefficient larger than zero represents a positive correlation, while Pearson correlation coefficient less than zero represents a negative correlation.

Regarding miRNAs in the plasma, miR-122 was negatively correlated with *hmgcr* and *cyp51* while miR-128 also showed negative correlations with *hmgcs*, all genes involved in cholesterol synthesis (*P<0*.*05*). Circulating miR-122 was also negatively correlated (*P<0*.*05*) with the gene expression of *srebp-2* which is a positive regulator of cholesterol synthesis, *abcg5* which is involved in cholesterol efflux and *acat2*. The plasma level of miR-223 was found to be negatively correlated with many genes involved in hepatic cholesterol efflux, including *ugt1a3*, *abcg5* and *abcg8*, but positively correlated with *abca1* (*P<0*.*05*). Moreover, miR-233 was also detected to negatively correlate with both *fas* and *gck* (*P<0*.*05*) (Table 6).

### Correlations between hepatic miRNAs and plasmatic miRNAs

Results showed that hepatic and circulating levels of miR-122 and miR-128 positively correlated with each other (*P<0*.*05*) whereas, no correlations were found for miR-1 and miR-223 (*P>0*.*05*) (Table 7).

**Table 7:** Correlations between miRNAs in the liver and plasma of rainbow trout.

Pearson value		Liver miR n = 30					Plasma miR n = 29				
		L-miR-1	L-miR-33a	L-miR-122	L-miR-128	L-miR-223	P-miR-1	P-miR-33a	P-miR-122	P-miR-128	P-miR-223
Liver miR n = 30	L-miR-1		0.596**	0.455*	0.597**	0.304	-0.074	0.319	0.275	0.397*	0.191
	L-miR-33a			0.508**	0.644**	0.245	0.106	0.355	0.171	0.336	0.022
	L-miR-122				0.890**	0.588**	-0.065	0.526**	0.182	0.595**	0.431*
	L-miR-128					0.713**	-0.102	0.372*	0.187	0.578**	0.419*
	L-miR-223						0.066	0.246	-0.041	0.390	0.548**
Plasma miR n = 29	P-miR-1							0.367	-0.225	-0.065	0.047
	P-miR-33a								0.355	0.708**	0.622**
	P-miR-122									0.459*	0.234
	P-miR-128										0.780**
	P-miR-223										

n = 30 for miRNAs in liver, n = 29 for miRNAs in plasma.

P-values

* *P*<0.05

** *P*<0.01.

Pearson correlation coefficient larger than zero represents a positive correlation, while Pearson correlation coefficient less than zero represents a negative correlation.

## Discussion

In the present study, rainbow trout was used as a research model in order to 1) understand the postprandial kinetic of parameters related to cholesterol metabolism, including miRNAs and 2) evaluate if circulating miRNAs could reflect the level of expression of genes in the liver and therefore be used as non-invasive biomarkers. For this purpose, we analyzed parameters at the phenotypic level (cholesterol, triglycerides and glucose in plasma) and transcriptional level (gene expressions in liver and miRNAs expressions in liver and plasma) at several time points after the last meal. Furthermore, the correlations between these parameters were investigated to assess the potential of miRNAs to be used as biomarkers in aquaculture regarding the development of alternative diets devoid of fishmeal and fish oil.

In this study, postprandial levels of cholesterol, triglycerides and glucose in plasma were only slightly affected by the meal. Originated from Acetyl-CoA, cholesterol is synthesized by about 20 enzymes and regulated by the SREBP pathway [68]. Following a meal, the hepatic expression kinetic of genes encoding four important enzymes responsible for cholesterol synthesis (*hmgcs*, *hmgcr*, *cyp51* and *dhcr7*), and their transcription factor *srebp-2* were quite similar. After a relatively stable period, the expression of these genes increased 16h after the meal before to come to basal level 24h after the meal, reflecting synchronous effects on cholesterol synthesis. Consistently with these results, significant positive correlations were observed between these genes, confirming their close cooperation during cholesterol synthesis.

*Abcg5*, *abcg8* and *ugt1a3* are genes encoding proteins involved in hepatic cholesterol efflux and elimination. ABCG5 and ABCG8 form an heterodimer that acts in the liver and intestine as a cholesterol transporter to excrete cholesterol into the bile [69], while UGT1A3 catalyzes its subsequent glucuronidation during bile acid synthesis [70]. The expression of these three genes showed the same postprandial pattern and their expression was also very similar to that of cholesterol synthetic genes. This suggests a close functional relation between ABCG5 and ABCG8 at the transcriptional level in rainbow trout. However, other genes involved in cholesterol efflux, such as, *cyp7a1*, *abca1* and *lxrα* were not significantly affected by feeding. Lxrα has been shown to act as a cholesterol sensor to regulate the expression of *cyp7a1*, *abcg5*, *abcg8* and *abca1* [71]. Accordingly, in this study, we found a significant correlation between *lxrα* and *cyp7a1* and *abcg5*in rainbow trout.

ACAT1 and ACAT2 are enzymes that catalyze the esterification of cholesterol to cholesterol ester for storage or transportation [72]. Although a peak of expression was recorded for these two genes 16h after the meal, *acat1* and *acat2* expressions did not correlate with each other. Nevertheless, they presented significant correlation with the expression of genes involved in cholesterol biosynthesis and *srebp-2*. This may be attributed to the fact that *acat2* is one of *srebp-2* target genes as previously reported in mouse [73] and plays important roles in cholesterol storage and transportation.

Among lipid metabolism, SREBP-1c is the major regulator of several genes related to fatty acid synthesis amongst which *fas* encodes for the key enzyme of fatty acid synthesis [74,75]. This is supported by the significant positive correlation found in the present study between *fas* and *srebp-1c* gene expression. Meanwhile, the positive correlation between these two genes and the four main genes involved in cholesterol synthesis also suggested a close relationship between fatty acid synthesis and cholesterol synthesis. GCK is known to play a major role in controlling glucose utilization in glycolytic pathways [76] and is also considered as the first enzyme of lipogenesis. The positive correlation between *gck* and cholesterol synthetic and efflux genes may suggest that cholesterol synthesis and efflux are processes requiring ATP which could be supplied by the glycolytic reaction catalyzed by GCK.

At posttranscriptional level, several miRNAs involved in cholesterol metabolism were detected both in liver and plasma and showed postprandial variations. Postprandial changes of miRNAs expression have already been demonstrated in trout liver [47] and skeletal muscle of Chinese perch (*Siniperca chuatsi*) [77]. The present study further indicates that circulating miRNAs levels also vary following trout feeding.

According to previous studies, the functional mechanisms of several miRNAs involved in cholesterol regulation are as follows: miR-1 may promote cellular cholesterol through LXRα suppression [59], miR-33a represses cholesterol efflux by targeting ABCG5, ABCG8, CYP7A1 and ABCA1 [60,78,79], miR-122 may increase some genes in cholesterol synthesis, though this mechanism of activation is still unknown [80], miR-128 increases cellular cholesterol by ABCA1 suppression and SREBP-2 promotion [63] and miR-223 reduces cellular cholesterol and promotes cholesterol efflux by repressing directly *hmgcs* and indirectly *abca1* and *cyp7a1*, respectively [64].

The hepatic expression of miR-1, miR-122 and miR-128 showed very similar evolution after the meal, reaching their highest and lowest expressions 12h and 16h after the meal, respectively. Compared with most genes analyzed in the present study, which reached their highest expression 16h after the meal, the expression of miR-1, miR-122 and miR-128 increased earlier. Although further investigations are needed to highlight a cause to effect relationship, our results suggest that these miRNAs may be involved in the regulation of these aforementioned genes.

The correlation found between miR-122 and cholesterol synthetic genes is in agreement with the effect of miR-122 on cholesterol biosynthesis determined in a previous study using antagomirs to inhibit miR-122 *in vivo* [80]. In mouse, it was also shown that miR-223 inhibited the cholesterol synthesis by the direct repression of *hmgcs* [64]. This is in agreement with our results showing that miR-223 negatively correlates with genes involved in cholesterol synthesis. It was also reported that the expression of *srebp-1*, *srebp-2*, *lxrα* and *abca1* was affected by miR-128 in mouse and human cell lines [63]. However, in our study, we did not observe any correlation between miR-128 and these genes in rainbow trout. The negative correlation between miR-122, miR-223 and miR-128 and genes involved in hepatic cholesterol metabolism suggest that these genes may be potential direct targets of miR-122, miR-223 and miR-128. Further analysis of the complementarity between 3’ untranslated region of the genes and seed sequences of the miRNAs are needed to establish this relation. Although none of the genes evaluated in the present study correlate with hepatic and plasma levels of miR-33a, miR-33a is the only miRNA that was strongly and positively correlated with plasma levels of cholesterol and triglycerides, suggesting a role of miR-33a in cholesterol accumulation in fish as previously shown in mammals [60,78].

For the first time, we demonstrate the presence of miRNAs (miR-1, miR-33a, miR-122, miR-128 and miR-223) in the plasma of rainbow trout. Based on mammalian studies, miRNAs present in the plasma may have several origins: the passive release from dead cells, an active secretion *via* microvesicles or an active secretion with RNA-binding proteins [19]. The potential roles of secreted miRNAs have been reported to be involved in the regulation of various physiological and pathological processes in mammals. For example, Mittelbrunn M. *et al*. proposed that miR-335 generated from T cell are transferred to antigen presenting cells and regulate its cellular target genes during immune response [81], suggesting that secreted miRNAs can be used as biomarkers to reflect target gene expression in cells. Despite the biological function and secretory mechanism of extracellular miRNAs are still unclear and remain controversial in mammals, the potential of miRNAs as biomarkers for cancer diagnosis and prognosis has already been verified. For example, miR-29a and miR-92a levels in plasma are significantly higher in patients with advanced-stage colorectal carcinoma than healthy controls [82]. Therefore, we further studied miRNAs in plasma and analyzed the correlations between circulating miRNAs and their hepatic counterparts as well as correlations between circulating miRNAs and genes involved in cholesterol metabolism in order to evaluate the potential of circulating miRNAs to be used as non-invasive biomarkers in rainbow trout.

Interestingly, we found a positive correlation between hepatic expression and plasma level of miR-128 and miR-223 suggesting that circulating miRNAs may be indicative of the expression of their hepatic counterparts in trout. As it is known that 1) circulating miRNAs may be released from either byproducts of cellular activity or selective miRNA export system [34], and 2) liver plays a central role in cholesterol homeostasis [83], the positive correlations that we have found for miR-128 and miR-223 levels between liver and plasma may reflect their active secretion from hepatocyte into plasma for posttranscriptional regulation of cholesterol metabolism in extrahepatic tissues. However, as miRNAs are widely expressed among tissues, their expression should be evaluated in other tissues to validate that liver exhibits the major contribution to circulating levels of miR-128 and miR-223.

As their hepatic counterparts, miR-122, miR-128 and miR-223 in plasma exhibit significant correlations with the expression of genes involved in cholesterol synthesis, efflux or esterification. The correlation between *gck* and miR-223 was also confirmed at circulating level. Once again, these results highlight the importance of miR-122 and miR-128 in cholesterol synthesis regulation and miR-223 in cholesterol efflux and glycolysis regulation. Circulating miR-223 was the only plasmatic miRNA that negatively correlates with plasma cholesterol level. This correlation was therefore consistent with the negative effect assigned to miR-223 on cholesterol homeostasis in mammals [64]. Altogether, our results strongly suggest that circulating miR-122, miR-128 and miR-223 could represent useful biomarkers of cholesterol and glucose metabolism in rainbow trout.

In conclusion, the present study demonstrates the presence of miRNAs in rainbow trout plasma and showed that hepatic expression of miRNAs and circulating miRNAs level are both subjected to postprandial regulations which could be attributed to nutritional aspects but also to circadian rhythm since analyses were performed over 24 hours. This study also indicates that correlations are found between hepatic and circulating levels of miRNAs as shown for miR-128 and miR-223 and between hepatic gene expression and liver or plasma miRNAs, as observed for miR-128 and genes involved in cholesterol synthesis. These results support the evidence that circulating miRNAs can reflect the level of expression of their tissue counterparts as well as the expression of genes in rainbow trout and highlight circulating miRNAs as promising non-invasive biomarkers in trout. However, correlations between circulating miRNAs and hepatic gene expression should not presume any direct or indirect regulatory relationship. Therefore, bioinformatics prediction of miRNA/target relationship as well as the implementation of functional studies based on miRNAs inhibitors or mimics or the development of 3’UTR luciferase reporter assays would be necessary to clearly demonstrate the regulatory role of these miRNAs on cholesterol metabolism. Further studies are now also necessary to better understand the mechanisms underlying miRNAs blood secretion and their subsequent molecular and physiological functions in fish. In particular, long term dietary trials with plant-based diets devoid of cholesterol should be conducted in order to improve our knowledges on the role of miRNAs in fish.

## Supporting information

S1 File Plasma metabolites, miRNA expression and gene expression data. Plasma metabolites excel sheet: postprandial levels of cholesterol (Chol), glucose and triglycerides (TG) in plasma of rainbow trout at 3h, 8h, 12h, 16h and 24h after the meal (n = 6 at each time point). Mir expression excel sheet: data of hepatic expression (Lmir) and plasma abundance (Pmir) of miR-1, miR-33, miR-122, miR-128 and miR-223 measured 3h, 8h, 12h, 16h and 24h after the meal (n = 6 at each time point). miRNA data are normalized with miR-39 used as exogenous spike miRNA. Gene expression excel sheet: data of hepatic expression of genes related to cholesterol synthesis (*hmgcr*, *hmgcs*, *cyp51*, *dhcr7* and *srebp2*), cholesterol efflux (*cyp7a1*, *ugt1a3*, *abcg5*, *abcg8*, *abca1* and *lxr*), cholesterol esterification (*acat1* and *acat2*) and lipogenesis (*fas*, *gck* and *srebp1*) measured 3h, 8h, 12h, 16h and 24h after the meal (n = 6 at each time point). miRNA data are normalized with with NORMA-Gene method.
(XLSX)
